# Sensitivity of SARS-CoV-2 Variants to Neutralization by Convalescent Sera and a VH3-30 Monoclonal Antibody

**DOI:** 10.3389/fimmu.2021.751584

**Published:** 2021-09-23

**Authors:** Shuai Yue, Zhirong Li, Yao Lin, Yang Yang, Mengqi Yuan, Zhiwei Pan, Li Hu, Leiqiong Gao, Jing Zhou, Jianfang Tang, Yifei Wang, Qin Tian, Yaxing Hao, Juan Wang, Qizhao Huang, Lifan Xu, Bo Zhu, Pinghuang Liu, Kai Deng, Li Wang, Lilin Ye, Xiangyu Chen

**Affiliations:** ^1^ Institute of Immunology, Third Military Medical University, Chongqing, China; ^2^ Department of Stomatology, Daping Hospital and Research Institute of Surgery, Third Military Medical University, Chongqing, China; ^3^ College of Veterinary Medicine, China Agricultural University, Beijing, China; ^4^ Department of Emergency Medicine, Southwest Hospital, Third Military Medical University, Chongqing, China; ^5^ Institute of Cancer, Xinqiao Hospital, Third Military Medical University, Chongqing, China; ^6^ Institute of Human Virology, Key Laboratory of Tropical Disease Control of Ministry of Education, Zhongshan School of Medicine, Sun Yat-sen University, Guangzhou, China; ^7^ Infectious Diseases Institute, Guangzhou Eighth People’s Hospital, Guangzhou Medical University, Guangzhou, China

**Keywords:** COVID-19, SARS-CoV-2 variants, neutralizing mAb, convalescent sera, antibody response

## Abstract

The severe acute respiratory syndrome coronavirus 2 (SARS-CoV-2) has caused a global pandemic of novel coronavirus disease (COVID-19). Though vaccines and neutralizing monoclonal antibodies (mAbs) have been developed to fight COVID-19 in the past year, one major concern is the emergence of SARS-CoV-2 variants of concern (VOCs). Indeed, SARS-CoV-2 VOCs such as B.1.1.7 (UK), B.1.351 (South Africa), P.1 (Brazil), and B.1.617.1 (India) now dominate the pandemic. Herein, we found that binding activity and neutralizing capacity of sera collected from convalescent patients in early 2020 for SARS-CoV-2 VOCs, but not non-VOC variants, were severely blunted. Furthermore, we observed evasion of SARS-CoV-2 VOCs from a VH3-30 mAb 32D4, which was proved to exhibit highly potential neutralization against wild-type (WT) SARS-CoV-2. Thus, these results indicated that SARS-CoV-2 VOCs might be able to spread in convalescent patients and even harbor resistance to medical countermeasures. New interventions against these SARS-CoV-2 VOCs are urgently needed.

## Introduction

As the causative agent of COVID-19, SARS-CoV-2 has caused a global pandemic with more than 211.28 million cases and 4.42 million fatalities as of August 24, 2021 (1). The SARS-CoV-2 utilizes its spike (S) protein, including the surface subunit S1 and the transmembrane subunit S2, for receptor binding and virus entry. Specifically, the S1 domain binds to the cellular receptor angiotensin-converting enzyme 2 (ACE2) *via* its receptor binding domain (RBD). The engagement of ACE2 with RBD further leads to the shedding of S1 subunit from S2 subunit, which promotes S2-mediated virus–host membrane fusion and virus entry (2, 3). Given the critical role of RBD protein in initiating SARS-CoV-2 infection, it becomes one primary target of neutralizing antibodies elicited by both natural infection and vaccination (4–6).

However, one major concern is the emergence of SARS-CoV-2 variants of concern (VOCs), in particular, with mutation(s) located in the RBD region (7, 8). These SARS-CoV-2 VOCs threaten efforts to contain the COVID-19 pandemic and include B.1.1.7 (N501Y in RBD) (9), B.1.351 (K417N, E484K, and N501Y in RBD) (10), P.1 (K417T, E484K and N501Y in RBD) (11), and B.1.617.1 (L452R and E484Q in RBD) (12). Indeed, these SARS-CoV-2 VOCs harbor transmission advantage over non-VOC variants and account more than 90% of currently sequenced SARS-CoV-2 viruses (8). To address the potential neutralization escape caused by these mutations in RBD, we analyzed the binding activity and neutralizing capacity of serum collected from a cohort of convalescent patients with different clinical symptoms in early 2020 against SARS-CoV-2 VOCs as well as non-VOC variants. In addition, we profiled the neutralizing capacity of one previously reported VH3-30 monoclonal antibody (mAb) against SARS-CoV-2 VOCs and non-VOC variants.

## Materials and Methods

### Human Samples

We enrolled a cohort of 28 convalescent COVID-19 patients with severe (*n* = 11), moderate (*n* = 9), and mild/asymptomatic (*n* = 8) symptoms upon being admitted to Guangzhou Eighth People’s Hospital. All COVID-19 patients were positive for SARS-CoV-2 virus RNA qPCR test upon hospital admission. COVID-19 patients were diagnosed as severe when meeting at least one of the following conditions: (1) RR ≥ 30/min, (2) PaO2/FiO2 ≤ 300 mmHg, (3) SpO2 ≤ 93%, and (4) imageological evidence of significant progress (>50%) in 24–48 h. COVID-19 patients with moderate symptoms were diagnosed by respiratory symptoms, fever, and imageological evidence of pneumonia. The mild COVID-19 patients were diagnosed by inapparent clinical symptoms and no imageological evidence of pneumonia. The asymptomatic COVID-19 patients were those who show no clinical symptoms. These patients were enrolled 15 to 32 days after symptom onset (January to March 2020); the medium age was 58 [43–64, interquartile range (IQR)] years; 60.7% were female; serum was collected from patients during convalescence and the time between symptom onset to serum sample collection was 23 (15–32, IQR) days. Healthy control subjects were six adult participants in the study. All the healthy control subjects were negative for SARS-CoV-2 virus RNA qPCR test upon blood-sampling collection (**Supplementary Table S1**). Sera were collected from blood without sodium citrate treatment and stored in aliquots at −80°C. The study received IRB approvals at Guangzhou Eighth People’s Hospital (KE202001134).

### Enzyme Linked Immunosorbent Assay

Fifty nanograms of SARS-CoV-2 RBD proteins of WT strain (Sino Biological, 40592-V08H), B.1.1.7 (Sino Biological, 40592-V08H82), P.1 (Sino Biological, 40592-V08H86), B.1.351 (Sino Biological, 40592-V08H85), and B.1.617.1 (Sino Biological, 40592-V08H88) as well as RBD proteins with point mutation such as W436R (Sino Biological, 40592-V08H9), F342L (Sino Biological, 40592-V08H6), V483A (Sino Biological, 40592-V08H5), K458R (Sino Biological, 40592-V08H7), A435S (Sino Biological, 40592-V08H4), N354D (Sino Biological, 40592-V08H2), G476S (Sino Biological, 40592-V08H8), and V367F (Sino Biological, 40592-V08H1) in 50 μl PBS per well was coated on ELISA plates overnight at 4°C. Then, the ELISA plates were blocked for 1 h with blocking buffer (5% FBS plus 0.05% Tween 20). Next, fivefold serially diluted mAbs or fivefold serially diluted patient sera were added to each well in 50 μl of blocking buffer for 1 h. After washing with PBST, the bound antibodies were incubated with anti-human IgG HRP detection antibody (Bioss Biotech) for 45 min, followed by washing with PBST and then reacting with TMB (Beyotime). The ELISA plates were allowed to react for 5 min and then stopped by 1 M H_2_SO_4_ stop buffer. The optical density (OD) value was determined at 450 nm. Concentration for 50% of maximal effect (EC_50_) was calculated by using nonlinear regression.

### ELISA-Based Receptor-Binding Inhibition Assay

Two hundred nanograms of hACE2 protein (Sino Biological, 10108-H05H) in 50 μl PBS per well was coated on ELISA plates overnight at 4°C. Then, the ELISA plates were blocked for 1 h with blocking buffer (5% FBS plus 0.05% Tween 20); meanwhile, threefold serial diluted mAbs or twofold diluted patient sera were incubated with 0.2 μg/ml SARS-CoV-2 RBD protein for 1 h. Then, the incubated mixtures were added to ELISA plates and allowed to develop for 1 h, followed by PBST washing and anti-His HRP antibody (Sino Biological, 105327-MM02T-H) incubating for 45 min. Next, the ELISA plates were washed with PBST and added with TMB (Beyotime). After 5 min, the ELISA plates were stopped and determined at 450 nm. The half maximal inhibitory concentration (IC_50_) was determined by using four-parameter logistic regression.

### SARS-CoV-2 Pseudovirus Neutralization Assay

For neutralization experiments, SARS-CoV-2 pseudotype particles were pre-incubated with serial diluted convalescent sera or mAbs for 1 h at 37°C. Then, hACE2-expressing HEK-293T (hACE2/293T) cells were incubated with the mixtures overnight and then cultured with fresh media. At 48 h after the mixture incubation, the luciferase activity of SARS-CoV-2 typed pseudovirus-infected hACE2/293T cells were measured by a luciferase reporter assay kit (Promega, E1910).

### Statistics

The SARS-CoV-2 RBD antibody titers, the virus neutralizing function of the sera belonging to patients, and the virus neutralizing function of mAb 32D4 were compared with the one-way ANOVA test. *p*-values less than 0.05 were defined as statistically significant. GraphPad Prism version 6.0 software was used for statistical analysis.

## Results

### Reduced Titer of Sera Antibodies Specific for SARS-CoV-2 VOC RBD in Individuals Recovered From WT SARS-CoV-2 Infection

**Figure 1:**
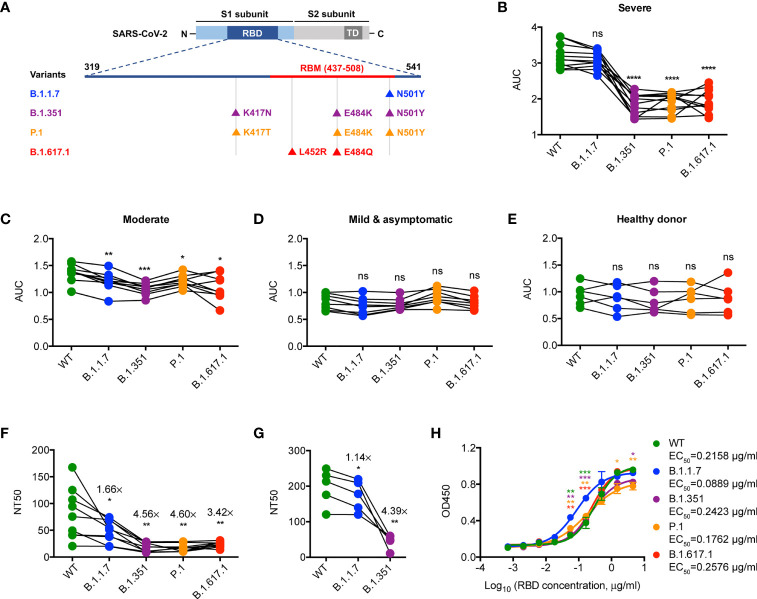
Neutralization of SARS-CoV-2 VOCs by convalescent sera. **(A)** Schematic diagram showing the location of mutations of SARS-CoV-2 VOCs in the context of RBD protein domain. RBD, receptor binding domain; RBM, receptor binding motif; TD, transmembrane domain. **(B–E)** ELISA binding assay of COVID-19 convalescent patient sera **(B–D)** or healthy donor sera **(E)** to ELISA plate coating of RBD proteins of SARS-CoV-2 and its mutated variants as indicated. AUC, area under the curve. **(F)** COVID-19 convalescent patient serum-mediated inhibition of indicated RBD proteins binding to ACE2 protein by ELISA. NT50, neutralizing titer 50. **(G)** COVID-19 convalescent patient serum-mediated neutralization of indicated SARS-CoV-2 pseudoviruses. NT50, neutralizing titer 50. **(H)** ELISA binding assay of ACE2 to indicated RBD proteins. EC_50_, concentration for 50% of maximal effect. The data are representative of at least two independent experiments. *p < 0.05, **p < 0.01, ***p < 0.001, and ****p < 0.0001. Not significant, ns. Error bars in **(H)** indicate SD.

Firstly, we examined the binding activity of antibodies that specifically bind to the RBD protein of WT SARS-CoV-2 strain and the mutated RBD proteins of SARS-CoV-2 VOCs (including B.1.1.7, B.1.351, P.1, and B.1.617.1) (**Figure 1A**) in the convalescent sera of WT SARS-CoV-2-infected patients (early 2020) by IgG ELISA. Notably, we found a significantly lower binding activity of antibodies specific for B.1.351, P.1, and B.1.617.1 RBDs but not B.1.1.7 RBD when compared to those of the WT one in the group of convalescent COVID-19 patients with severe illness (**Figure 1B**). This feature was less pronounced when extended to convalescent COVID-19 patients with moderate or mild/asymptomatic illness (**Figures 1C–E**), which might be due to the suboptimal tonic RBD-specific antibodies in these patients (4, 13). Consistently, binding ability of convalescent sera from COVID-19 patients with severe illness against SARS-CoV-2 VOCs, albeit blunted, was superior to those of COVID-19 patients with moderate or mild/asymptomatic illness (**Supplementary Figures S1A–E**). Therefore, these results suggest a crucial role of residues N501, E484, L452, and K417 in epitope regions of high-affinity antibodies specific for SARS-CoV-2 RBD.

### Reduced Neutralization Against SARS-CoV-2 VOCs by Convalescent Sera Elicited by WT SARS-CoV-2 Infection

We then assessed the neutralizing capacity of convalescent sera from WT SARS-CoV-2-infected patients with severe illness by ELISA-based RBD-ACE2 binding inhibition assays and pseudovirus neutralization assays as previously described (4, 14). Neutralization against B.1.1.7 by convalescent sera was slightly less efficient as compared to that against WT (**Figures 1F, G**), which might be due to higher ACE2 binding ability observed in B.1.1.7 (**Figure 1H**). However, the neutralizing potency of convalescent sera against B.1.351, P.1, and B.1.617.1 was significantly reduced when compared to that against WT (**Figures 1F, G**). Given the comparable ACE2 binding ability between WT and VOCs (including B.1.351, P.1, and B.1.617.1) (**Figure 1H**), the noticeable resistance of these VOCs to convalescent sera was likely caused by the lack of binding ability to the RBD with E484K, L452R, and K417N/T mutations (**Figure 1B**). Thus, SARS-CoV-2 VOCs may partially evade the neutralization by antibodies elicited by the WT strain infection.

### Similar Binding Activity and Neutralizing Capacity of Convalescent Sera for SARS-CoV-2 Non-VOC Variants

**Figure 2:**
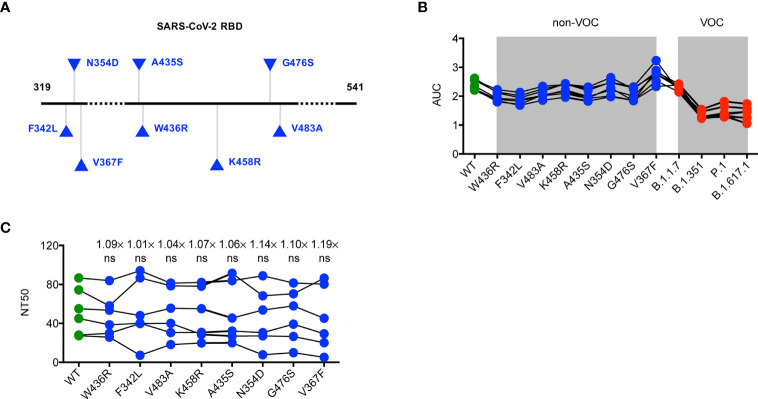
Neutralization of SARS-CoV-2 non-VOC variants by convalescent sera. **(A)** Schematic diagram presenting the location of mutations of non-VOC variants in the context of RBD protein domain. **(B)** ELISA binding assay of sera originated from COVID-19 patients with severe illness to ELISA plate coating of RBD proteins of WT RBD and its mutated variants as indicated. **(C)** COVID-19 convalescent patient serum-mediated inhibition of indicated RBD proteins binding to ACE2 protein by ELISA. The data are representative of at least two independent experiments. Not significant, ns.

Considering a substantial transmission disadvantage of SARS-CoV-2 non-VOC variants in the COVID-19 pandemic (15), we next sought to analyze the neutralizing potency of convalescent sera against non-VOC variants with a different RBD mutation, including F342L, N354D, V367F, A435S, W436R, K458R, G476S, and V483A (**Figure 2A**). We found it of particular interest that the binding and neutralizing ability of specific antibodies in convalescent serum for the RBD of SARS-CoV-2 non-VOC were not weaker than those for the WT RBD (**Figures 2B, C**). This finding indicates minimal influences of these mutations to the neutralizing activity of SARS-CoV-2 RBD-targeted mAbs and also excludes the possibility that the SARS-CoV-2 pandemic shifts to these non-VOC variants.

### Neutralization Sensitivity of a VH3-30 mAb 32D4 to SARS-CoV-2 Variants

**Figure 3:**
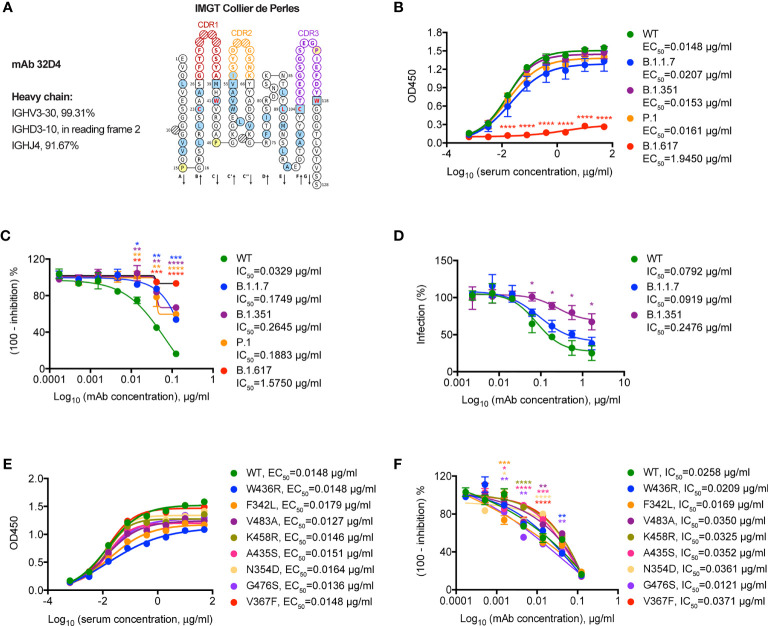
Neutralization of SARS-CoV-2 variants by a VH3-30 mAb 32D4. **(A)** IMGT Collier de Perles for 32D4. **(B)** ELISA binding assay of mAb 32D4 to RBD proteins of WT and VOCs. EC_50_, concentration for 50% of maximal effect. **(C)** ELISA analysis of mAb 32D4-mediated inhibition of WT and VOC RBD proteins binding to ACE2 protein. IC_50_, half maximal inhibitory concentration. **(D)** 32D4-mediated neutralization of indicated SARS-CoV-2 pseudoviruses. IC_50_, half maximal inhibitory concentration. **(E)** ELISA binding assay of mAb 32D4 to RBD proteins of WT and non-VOC variants. EC_50_, concentration for 50% of maximal effect. **(F)** ELISA analysis of mAb 32D4-mediated inhibition of WT and non-VOC RBD proteins binding to ACE2 protein. IC_50_, half maximal inhibitory concentration. *p < 0.05, **p < 0.01, ***p < 0.001, and ****p < 0.0001. The data are representative of at least two independent experiments. Error bars in **(B–D, F)** indicate SD.

Finally, we set out to determine the neutralizing capacity of 32D4 mAb on these SARS-CoV-2 variants. The 32D4 mAb, isolated from memory B cells of WT SARS-CoV-2-infected patients, is one of the first identified human neutralizing mAbs that target SARS-CoV-2 RBD (14). As analyzed by IMGT (16), the 32D4 mAb is encoded by the IGHV3-30 gene (**Figure 3A**), which is one of the most enriched IGHV genes used by RBD-targeting antibodies and thus characterizes one binding mode of RBD-targeting antibodies (17). As shown, the 32D4 mAb showed high binding affinity for SARS-CoV-2 VOCs, with EC_50_ values of 0.0207 μg/ml for B.1.1.7, 0.0153 μg/ml for B.1.351, and 0.0161 μg/ml for P.1 (**Figure 3B**). However, the binding affinity of 32D4 for B.1.617.1 was severely blunted and the EC_50_ value was increased to 1.9450 μg/ml (**Figure 3B**), indicative of a key role of the residue L452 for the 32D4 binding epitope. Besides, 32D4 was less effective in inhibiting B.1.1.7, B.1.351, and P.1 to engage with ACE2 as compared to the WT one and completely failed to block interaction between B.1.617.1 and ACE2 as evidenced by functional ELISA assays (**Figure 3C**). Consistently, neutralization of mAb 32D4 against SARS-CoV-2 B.1.351 pseudoviruses was also blunted (**Figure 3D**). Along with our finding, recent studies also found that the neutralizing activity of several mAbs, including those being approved or in the late clinical stage, was abolished by SARS-CoV-2 VOCs (8, 18–20). In contrast, the binding and neutralizing ability of 32D4 mAb for SARS-CoV-2 non-VOC variants were largely unaffected (**Figures 3E, F**). Thus, these results suggest that neutralizing mAb targeting the SARS-CoV-2 WT protein sequence might be re-examined whether they are suitable as prophylaxis or treatment for individuals infected with SARS-CoV-2 VOCs.

## Discussion

The circulating SARS-CoV-2 VOCs, including B.1.1.7, B.1.351, P.1, and B.1.617.1, have taken a major toll on the global control of the COVID-19 pandemic. Indeed, accumulating evidence suggested reduced neutralization against SARS-CoV-2 VOCs by convalescent sera elicited by SARS-CoV-2 D614G variant (20, 21) or SARS-CoV-2 B.1.1.117 variant (22), sera from mRNA-1273- or BNT162b2-vaccinated individuals (5, 20, 21, 23), and FDA-approved neutralizing mAbs (8, 18). In the study, we also found attenuated neutralization capacity against SARS-CoV-2 VOCs, especially B.1.351, P.1, and B.1.617.1, by sera collected from convalescent patients in the early 2020 or by a VH3-30 mAb 32D4 isolated from the memory B cells of these convalescent patients.

Neutralization resistance of B.1.1.7 to convalescent sera and mAb 32D4 was not noticeable as compared to that of other VOCs in our study. This dichotomous neutralization resistance was also reported by other studies (24, 25) and seems paradoxical to the increased affinity between the B.1.1.7 RBD with a single N501Y mutation and ACE2 observed in our study and previous studies (17, 26). However, we and another group (23) found that SARS-CoV-2-specific mAbs show partial or complete loss of binding to RBD with E484K substitution but not N501Y substitution. Besides, diminished neutralization capacity of convalescent sera and neutralizing mAbs was mainly caused by single mutation at residue E484 but not N501 (18, 23, 25). Thus, RBD E484 residue is a crucial binding site for mAbs and VOCs with mutation at E484 (E484K for B.1.351 and P.1; E484Q for B.1.617.1) show enhanced neutralization resistance.

The VH3-30 gene is one of the most-enriched IGHV genes used by RBD-targeting neutralizing mAbs elicited by natural infection (17, 27) and vaccination (28). SARS-CoV-2-specific neutralizing mAbs with VH3-30 gene, exemplified by REGN10987 (17, 29), C002 (17), and 32D4 in the study, are characterized by a similar binding mode to some extent (17, 30) and consequent mutational escape of SARS-CoV-2 variants. REGN10987 is suggested to be escaped by SARS-CoV-2 variants with mutations ranging from N439 to N453 within RBD, especially K444Q and V445A (31). The L452 residue is a key recognizing site for C002 (17). Here, we also found the losing binding and neutralization of 32D4 to B.1.617.1 variant with L452R substitution. In addition to B.1.617.1, other VOCs (e.g., B.1.1.7, B.1.351, and P.1) also partially escape neutralization by 32D4. The mechanism underlying the escape of VOCs to 32D4-mediated neutralization awaits further structural analysis.

Convalescent plasma or sera transfusion has been highlighted as a promising therapy in fighting newly emerged viral infections. Indeed, transfusion of convalescent plasma harvested from recovered COVID-19 patients is reported to be beneficial in treating critically ill patients with SARS-CoV-2 infection (32–35). Given the neutralization resistance of SARS-CoV-2 VOCs to convalescent sera collected from individuals infected with WT SARS-CoV-2 infection in early 2020, transfusion of these convalescent sera might not be suitable in treating COVID-19 patients infected with SARS-CoV-2 VOCs. Consistently, Cele et al. found that the B.1.351 variant was poorly neutralized by plasma from individuals infected with non-VOC B.1.1.117 (22). By contrast, cross-neutralization of non-VOC B.1.1.117 by plasma from those infected with B.1.351 was more effective (22). These results suggest the potential neutralization of plasma from SARS-CoV-2 VOC-infected individuals to WT, other VOCs, and non-VOC variants, which awaits further investigation.

As with other RNA viruses such as influenza and HIV, SARS-CoV-2 is also characterized by antigenic drift (17). In addition to E484K, L452R, and K417N/T mutations, numerous RBD mutations (including F342L, N354D, V367F, A435S, W436R, K458R, G476S, and V483A) have also been detected in non-VOC variants (36, 37). Though these RBD mutants show significantly increased affinity to hACE2 (36, 37), we found largely unaffected neutralizing potencies of convalescent sera and mAb 32D4 against SARS-CoV-2 variants with relevant RBD mutation. These results might explain the rare cases of these non-VOC variants during the COVID-19 pandemic and further indicated a crosstalk between human host immune pressure and SARS-CoV-2 variant selection.

Taken together, our study presents the comparison of sensitivity of SARS-CoV-2 VOC and non-VOC variants to neutralization by convalescent sera and a VH3-30 mAb from convalescent patients in the early 2020. Although these results are based on functional ELISA assays and pseudovirus assays and await confirmation with authentic SARS-CoV-2, the ELISA/pseudovirus assays have been proven to be free of biosafety issue but as reliable as the canonical plaque assay with authentic SARS-CoV-2 (4, 38–41). The results suggest that SARS-CoV-2 VOCs might be able to spread in convalescent patients and even harbor resistance to medical countermeasures. Indeed, we observed evasion of SARS-CoV-2 VOCs from the 32D4 mAb, which was proved to exhibit highly potential neutralization against WT SARS-CoV-2. Thus, containment of these SARS-CoV-2 VOCs by medical interventions (e.g., next-generation vaccines, pan-neutralizing mAbs) is in urgent need.

## Data Availability Statement

The original contributions presented in the study are included in the article/**Supplementary Material**. Further inquiries can be directed to the corresponding authors.

## Ethics Statement

The studies involving human participants were reviewed and approved by Guangzhou Eighth People’s Hospital (KE202001134). The patients/participants provided their written informed consent to participate in this study.

## Author Contributions

SY, ZL, YL, YY, and MY performed the experiments. ZP, LH, LG, JZ, JT, YW, QT, YH, JW, QH, and LX assisted in processing patient samples. XC designed the study, analyzed the data, and wrote the paper with LY, LW, KD, PL, SY, and BZ. XC and LY supervised the study. All authors contributed to the article and approved the submitted version.

## Funding

This work was supported by grants from the National Natural Science Fund for Distinguished Young Scholars (No. 31825011 to LY) and the National Natural Science Foundation of China (No. 31900643 to QH).

## Conflict of Interest

The authors declare that the research was conducted in the absence of any commercial or financial relationships that could be construed as a potential conflict of interest.

## Publisher’s Note

All claims expressed in this article are solely those of the authors and do not necessarily represent those of their affiliated organizations, or those of the publisher, the editors and the reviewers. Any product that may be evaluated in this article, or claim that may be made by its manufacturer, is not guaranteed or endorsed by the publisher.

## Supplementary Material

Supplementary Figure 1 Antibody responses to RBD proteins of WT and VOCs in patients recovered from different COVID-19 illness. **(A–E)** ELISA binding assay of COVID-19 convalescent patient sera and healthy donor sera to ELISA plate coating of RBD proteins of WT **(A)**, B.1.1.7 **(B)**, B.1.351 **(C)**, P.1 **(D)** and B.1.617.1 **(E)**. AUC, area under the curve. **P* < 0.05, ***P* < 0.01, ****P* < 0.001 and *****P* < 0.0001. Not significant, ns. Error bars in **(A–E)** indicate SEM.
