# Supplementary figures and images for: Sensitivity of SARS-CoV-2 Variants to Neutralization by Convalescent Sera and a VH3-30 Monoclonal Antibody
Source: Front Immunol. 2021 Sep 23;12:751584. doi: 10.3389/fimmu.2021.751584 (PMC8495157; doi:10.3389/fimmu.2021.751584)

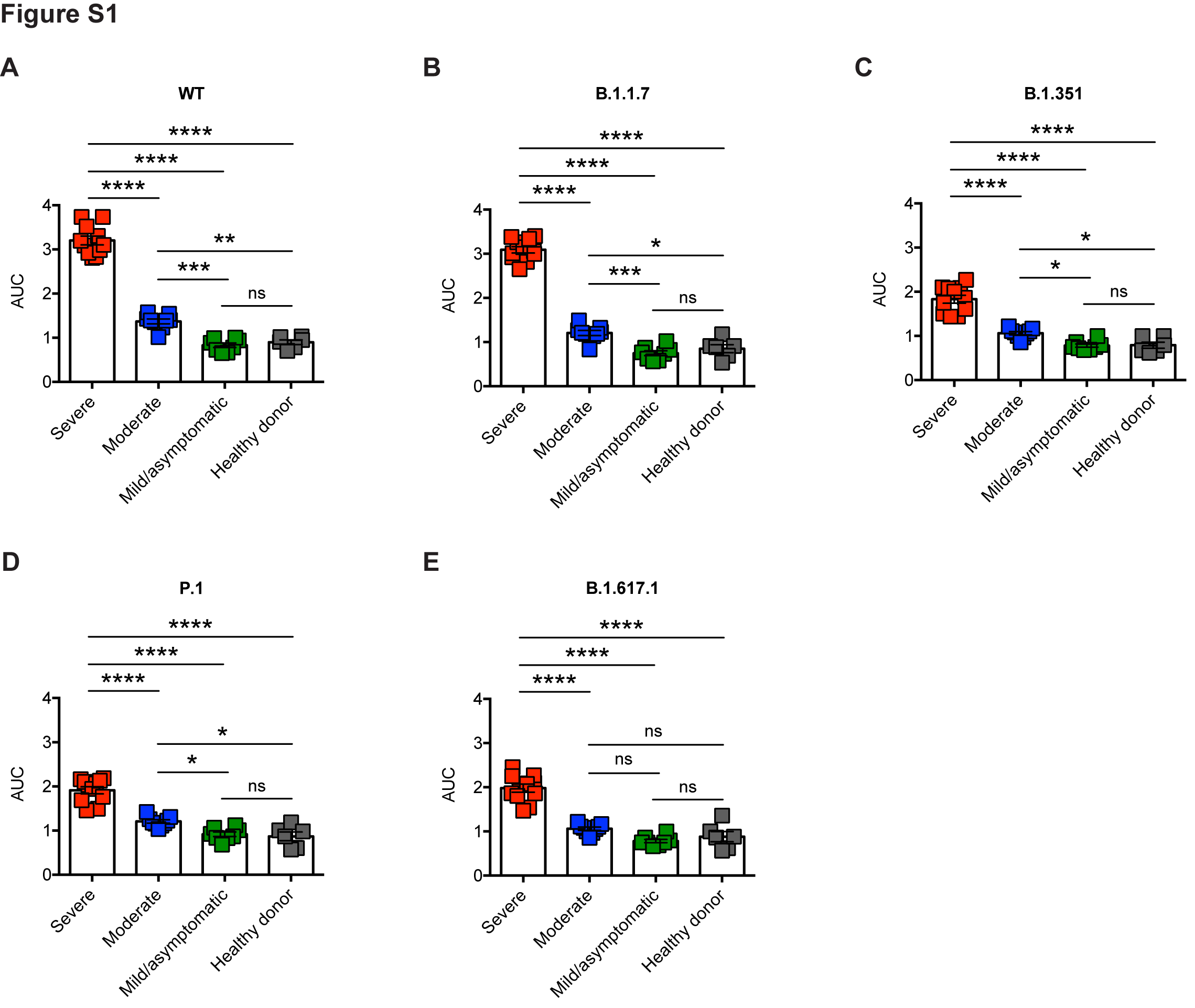

Supplement: Supplementary Figure 1 — Antibody responses to RBD proteins of WT and VOCs in patients recovered from different COVID-19 illness. (A–E) ELISA binding assay of COVID-19 convalescent patient sera and healthy donor sera to ELISA plate coating of RBD proteins of WT (A), B.1.1.7 (B), B.1.351 (C), P.1 (D) and B.1.617.1 (E). AUC, area under the curve. *P < 0.05, **P < 0.01, ***P < 0.001 and ****P < 0.0001. Not significant, ns. Error bars in (A–E) indicate SEM. [file Image_1.tif]
