# Supplementary material for: Sensitivity of SARS-CoV-2 Variants to Neutralization by Convalescent Sera and a VH3-30 Monoclonal Antibody
Source: Front Immunol. 2021 Sep 23;12:751584. doi: 10.3389/fimmu.2021.751584 (PMC8495157; doi:10.3389/fimmu.2021.751584)
Supplement: Supplementary file 2 [file Table_1.pdf]

Table S1

	All (n=28)	Severe (n=11)	Moderate (n=9)	Mild/asymptomatic (n=8)	Healthy controls (n=6)
**Age-years (median, IQR)**	58 (43-64)	64 (60-71)	58 (48-63)	31 (18-46)	31 (29-33)
**Gender**					
**Female**	17 (60.7%)	4 (36.4%)	7 (77.8%)	6 (75.0%)	2 (33.3%)
**Male**	11 (39.3%)	7 (63.6%)	2 (22.2%)	2 (25.0%)	4 (66.7%)
**Onset of symptom to sample collection-days (median, IQR)**	23 (15-32)	16 (13-30)	28 (20-33)	31 (18-46)	/
**Hospital admission date (medium, IQR)**	2020/1/28 (2020/1/24 to 2020/3/15)	2020/1/27 (2020/1/24 to 2020/1/31)	2020/1/25 (2020/1/19 to 2020/1/27)	2020/3/27 (2020/3/25 to 2020/4/28)	/
